# Achieving abiotic stress tolerance in plants through antioxidative defense mechanisms

**DOI:** 10.3389/fpls.2023.1110622

**Published:** 2023-06-02

**Authors:** Neelam Mishra, Chenkai Jiang, Lin Chen, Abhirup Paul, Archita Chatterjee, Guoxin Shen

**Affiliations:** ^1^ Department of Botany, St. Joseph’s University, Bangalore, KA, India; ^2^ Institute of Sericulture and Tea, Zhejiang Academy of Agricultural Sciences, Hangzhou, Zhejiang, China; ^3^ Independent Researcher, Bangalore, KA, India

**Keywords:** abiotic stress, reactive oxygen species, enzymatic antioxidants, non-enzymatic antioxidants, stress signaling

## Abstract

Climate change has increased the overall impact of abiotic stress conditions such as drought, salinity, and extreme temperatures on plants. Abiotic stress adversely affects the growth, development, crop yield, and productivity of plants. When plants are subjected to various environmental stress conditions, the balance between the production of reactive oxygen species and its detoxification through antioxidant mechanisms is disturbed. The extent of disturbance depends on the severity, intensity, and duration of abiotic stress. The equilibrium between the production and elimination of reactive oxygen species is maintained due to both enzymatic and non-enzymatic antioxidative defense mechanisms. Non-enzymatic antioxidants include both lipid-soluble (α-tocopherol and β-carotene) and water-soluble (glutathione, ascorbate, etc.) antioxidants. Ascorbate peroxidase (APX), superoxide dismutase (SOD), catalase (CAT), and glutathione reductase (GR) are major enzymatic antioxidants that are essential for ROS homeostasis. In this review, we intend to discuss various antioxidative defense approaches used to improve abiotic stress tolerance in plants and the mechanism of action of the genes or enzymes involved.

## Introduction

Abiotic stress, namely drought, temperature extremes (such as heat, chilling, and freezing), salinity, heavy metals, and UV radiation, can decrease plant growth and productivity by more than 50% (Khan et al., 2015; Minhas et al., 2017). Due to global warming and varying climatic conditions, abiotic stresses have become more severe and unpredictable. Furthermore, sustainable food production is also at risk due to the reduction in the availability of land for agriculture and the increasing global population (Dubey et al., 2020).

**Figure 1:**
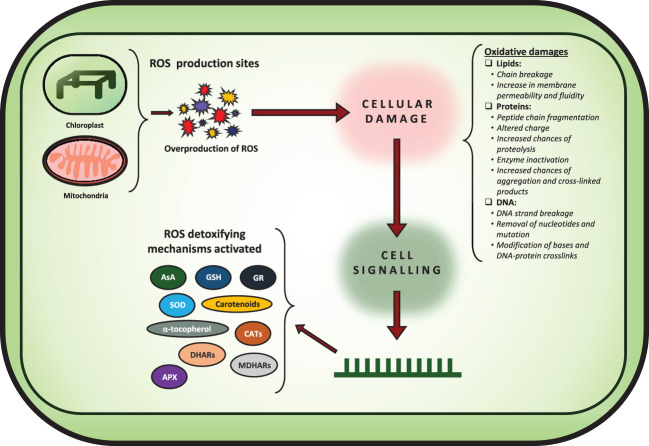
Figure showing how overproduction of ROS leads to various oxidative damages affecting the cellular machinery. Furthermore, the various ROS detoxifying mechanisms are shown as well.

Abiotic stresses significantly limit plant productivity by disturbing cellular biochemistry and physiology via overproduction of reactive oxygen species (ROS) (Apel and Hirt, 2004). ROS are known to play a dual role in plant growth and development as both signaling molecules and harmful agents that can cause oxidative damage to plant tissues (**Figure 1**). Excessive ROS accumulation due to the absence of ROS redox homeostasis can have detrimental effects on plant genetic materials, such as DNA, RNA, and proteins, leading to mutations, chromosomal aberrations, and even cell death (Apel and Hirt, 2004; Nakano et al., 2006; Mittler et al., 2011; Hussain et al., 2020). ROS such as hydrogen peroxide (H_2_O_2_), superoxide radical (O_2_^•−^), hydroxyl radical (OH^•^) and singlet oxygen (^1^O_2_), that are produced due to excitation or incomplete reduction of molecular oxygen are harmful byproducts of normal cellular metabolism in aerobic organisms (Mittler et al., 2011).

Plants have evolved various mechanisms to cope with oxidative stress, including the production of antioxidants and the activation of stress response pathways. Several transcription factors, including WRKY and NAC, have been identified as key regulators of ROS signaling and stress response pathways (Zhu, 2016; Zhang et al., 2017; Wang et al., 2021). Studies have also shown that epigenetic modifications, such as DNA methylation and histone acetylation, can modulate the expression of stress-responsive genes and enhance plant stress tolerance (Jaleel et al., 2007; Gill and Tuteja, 2010). Plants are endowed with a variety of enzymatic and non-enzymatic antioxidants that help them scavenge excess ROS and thereby play a crucial role in ROS homeostasis. Antioxidant enzymes in plants include superoxide dismutase (SOD), ascorbate peroxidase (APX), catalase (CAT), glutathione peroxidase (GPX), monodehydroascorbate reductase (MDHAR), dehydroascorbate reductase (DHAR), glutathione reductase (GR), and glutathione S-transferase (GST) (Mittler et al., 2011). Non-enzymatic antioxidants include glutathione, ascorbate, tocopherols, carotenoids, and flavonoids. Alternatively, excess generation of ROS can be prevented via manipulation of alternative oxidase (AOX) enzymes that can prevent the leakage of electrons to oxygen (Jaleel et al., 2007). In this review, we provide an overview of ROS regulation by enzymatic and non-enzymatic antioxidants. Furthermore, we discuss the improvement of abiotic stress tolerance through ROS regulation by means of antioxidants.

## Non- enzymatic antioxidants

Non-enzymatic antioxidants consist of low-molecular-weight compounds that are further categorized into water-soluble antioxidants, such as ascorbic acid and glutathione, and lipophilic antioxidants, such as tocopherols and β-carotenes. Ascorbic acid is one of the most comprehensively studied antioxidants and is present in the majority of plant cell types, organelles, and apoplasts (Gill and Tuteja, 2010; Dumanović et al., 2021). Mitochondria are the major site for ascorbate (AsA) synthesis, and ascorbate is transported through a proton-electrochemical gradient or facilitated diffusion to other cellular compartments (Gill and Tuteja, 2010). AsA is involved in the regulation of growth, metabolism, and differentiation in plants, alongside being known to scavenge free radicals and prevent oxidative damage caused by abiotic stress. In the ascorbate–glutathione (AsA-GSH) cycle, two molecules of AsA are utilized by ascorbate peroxidase (APX) to reduce hydrogen peroxide to water, generating monodehydroascorbate (MDHA) (a radical with a short lifespan). MDHA can further disproportionate into dehydroascorbate (DHA) and AsA. Also, AsA provides membrane protection by directly scavenging free radicals of oxygen and regenerates tocopherol from tocopheroxyl radical. Enhanced tolerance to ozone, salt, and polyethylene glycol was observed in tobacco when the MDHAR gene of *Arabidopsis thaliana* was overexpressed in tobacco (Dumanović et al., 2021). Increased AsA content was found in *Picea aspertata* seedlings when exposed to light and drought stress and in *Cassia auriculata* seedlings under UV-B stress (Eltayeb et al., 2007; Matos et al., 2022).

Tocopherols have a unique ability to quench singlet oxygen by a charge transfer mechanism. Out of all the four isomers (α, β, γ, and δ), the most biologically active is the α isomer. It is one of the most abundant antioxidants in the chloroplast membranes and is known to protect plants against photo-oxidative damage. The concentration of tocopherol increases under drought stress and high light stress, and it protects plants against oxidative damage. α-tocopherols are known to prevent the chain propagation step in lipid auto-oxidation and effectively scavenge and quench various ROS and lipid oxidation products, stabilize membranes, and regulate signal transduction (Peñuelas and Munné-Bosch, 2005; Agarwal, 2007; Pourcel et al., 2007; Shao et al., 2007; Yang et al., 2008; Bagheri et al., 2017; Kumar et al., 2020).

Glutathione, a tripeptide, occurs primarily as the reduced form (GSH), and its concentration is highest in chloroplasts (1-4 mM) in comparison to other organelles (Hajam et al., 2023). It has the ability to regenerate another powerful antioxidant, i.e., ascorbic acid via the AsA-GSH cycle (Hajam et al., 2023). Isoprenoids, such as carotenoids and tocopherols, play an important role in photoprotection as well as the response to cadmium stress (Nagajyoti et al., 2010; Nareshkumar et al., 2015). Carotenoids have the ability to delocalize unpaired electrons due to the presence of a conjugated double-bonded structure (Nareshkumar et al., 2015). They are responsible for quenching singlet oxygen without degradation and are known to chemically react with free radicals such as peroxyl (ROO^•^), hydroxyl (^•^OH), and superoxide radicals (O_2_^•−^) (Bagheri et al., 2017). Another commonly occurring antioxidant, polyphenols, is known to possess free radical scavenging activity. Phenolic compounds react with peroxyl radicals and form phenoxy radical intermediates that are rather stable and unreactive (Bagheri et al., 2017). These function as terminators of free radical chains and as chelators of redox-active metal.

## Enzymatic antioxidants

### Superoxide dismutase (SOD) [EC 1.15.1.1]

#### Overview and types

**Figure 2:**
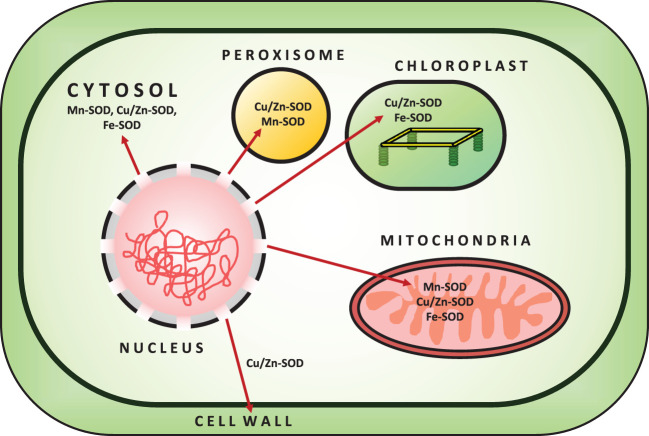
Localization of SOD enzymes in the Plant Cell.

SOD (EC 1.15.1.1) is a metalloenzyme and one of the most effective components of a plant cell’s antioxidant defense system against ROS toxicity. It catalyzes the conversion of O_2_^•−^ to O_2_ and H_2_O_2_. Initially, SOD was isolated as a green Cu-protein from bovine blood and was thought to be involved in Cu storage (Mittler, 2002). SOD is divided into three isoenzymes based on the presence of metal cofactors (Cu/Zn/Mn/Fe) at the active site (Alscher et al., 2002). All oxygen-metabolizing cells, as well as all subcellular compartments (such as chloroplasts, mitochondria, nuclei, peroxisomes, cytoplasm, and apoplasts), are assumed to contain SODs (Fink and Scandalios, 2002; Mahanty et al., 2012). Based on their localization, structure, and functions in plants, SOD can be classified into three isoenzymes, namely Cu/Zn-SOD, Mn-SOD, and Fe-SOD (Corpas et al., 2006) (**Figure 2**; **Table 1**). Cu/Zn-SOD has been found in cytosol, chloroplasts, and peroxisomes. Mn-SOD is found primarily in mitochondria, but also in peroxisomes and apoplasts (Pan et al., 2006). Fe-SOD is found primarily in chloroplasts and to a lesser extent in peroxisomes and apoplasts. A fourth group of SOD isoenzyme, Ni (II/III) at the active site (Ni-SOD), has also been discovered (Mahanty et al., 2012). Nonetheless, all SOD isoforms (Cu/Zn-SOD, Mn-SOD, Fe-SOD) are nuclear-coded and are transported to their organellar locations via NH_2_-terminal targeting sequences when necessary (Sharma et al., 2012). Further studies have revealed that Mn and Fe-SODs are found in both prokaryotes and eukaryotes, whereas Cu/Zn-SODs are primarily found in eukaryotes. Fe-SODs exist in homodimeric and tetrameric forms. They are resistant to KCN but not to H_2_O_2_. Similarly, Mn-SOD is homodimeric and homotetrameric, and it is resistant to both KCN and H_2_O_2_, whereas Cu/Zn-SOD is activated by both inhibitors (Gepstein and Glick, 2013). Furthermore, hormones such as abscisic acid and multifunctional signaling molecules such as melatonin regulate SOD activity via gene expression, like other antioxidant enzymes (Tuteja and Gill, 2013; Khan et al., 2020).

**Table 1:** Types of SOD, subcellular location and sensitivity.

SOD Isoenzymes	Structure	Species	Sensitivity	Subcellular localization
Cu/Zn-SOD	Homodimeric and Homotetrameric	*Pisum sativum, Zea mays, Spinacia oleracea, Avena sativa, Citrillus lanatus*	H_2_O_2_ and KCN	Cytosol, Chloroplast, Peroxisome Mitochondria
Mn-SOD	Homodimeric and Homotetrameric	*Vigna mungo, Zea mays, Pisum sativum, Spinacia oleracea, Nicotiana plumbaginifolia*	Not to H_2_O_2_ and KCN	Mitochondria Peroxisome Cytosol
Fe-SOD	Homodimeric and tetrameric	*Arabidopsis thaliana, Glycine max, Oryza sativa, Gingko biloba, Zea mays, Brassica campestris*	H_2_O_2_ but not to KCN	Cytosol Chloroplast Mitochondria

#### Response of SOD to abiotic stress

Abiotic stresses such as heat, cold, drought, salinity, and chemical contaminants are major factors limiting agricultural productivity around the world. As a result, understanding plant responses to these abiotic stresses has become a prerequisite for developing crop plants that can withstand abiotic stresses (Gepstein and Glick, 2013). Nonetheless, SODs are the first line of defense against abiotic stress because they are an important component of plant defense machinery. Due to stress, ROS and its reaction products increase, and dismutation of O_2_ is catalyzed by SODs into hydrogen peroxide (H_2_O_2_) and oxygen (O_2_). The impact of all SODs on the direct or indirect metabolism of various ROS has been confirmed (Mittler, 2006). Salinity is one of the most important abiotic stress factors that can affect the quality and yield of crops (Mittler, 2006). Although many plants have the ability to tolerate high salinity levels, most plants are susceptible to salt stress and show decreased photosynthetic activity or morphological disorders under such conditions (Sheokand et al., 2008; Szepesi et al., 2008; Zsigmond et al., 2012; Duque et al., 2013). Salinity causes an increase in ROS generation and alters the activity of ROS-scavenging enzymes. In certain plants, salt stress causes an increase in total SOD activity, while in others, no change or reduction in SOD activity was observed under abiotic stress. For example, under salt stress, increased numbers of leaves were seen in *Cicer arietinum*, *Beta vulgaris*, and *Brassica juncea* due to SOD activity, while a decrease in the number of leaves was observed in *Vigna unguiculata* (Hernandez et al., 2006; Sheokand et al., 2008; Kumar et al., 2013; Rasool et al., 2013). Furthermore, increased SOD activity under salt stress was reported in *Arabidopsis thaliana* and *Nicotiana tobacum* seedlings (Zsigmond et al., 2012; Lee et al., 2013). The significant differences in SOD activities in various plants under salt stress are evidence of its functioning at the inter-specific or intra-specific level. The effect of Cu/Zn-SOD was increased in the salt-tolerant and sensitive cultivars of Oryza sativa in response to salinity (Mishra et al., 2013). While studying the long-term effects of salt stress in two salt-tolerant lines (Kharchia65, KRL19) and two salt-sensitive lines (HD2009, HD2687) of *Triticum aestivum*, higher levels of SOD activity were observed in Kharchia65 when compared to HD2687 (Sairam et al., 2005). These inconsistencies show that SOD activity is influenced by a variety of factors, including the type of plant species (tolerant or sensitive), the intensity and duration of stress, and the plant organ used for the assays. Mn-SOD activity is found substantially in the cytosolic fraction. On the other hand, Kharchia65 had enhanced Mn-SOD activity in both fractions, which increases with salt stress. The activity of cytosolic Mn-SOD was very low, implying that it plays a minor role in scavenging salinity-induced O_2_ free-radical generation. Another isozyme, Cu/Zn-SOD, was found in all three cell compartments, with the chloroplast having the highest concentration, followed by mitochondria and cytosol. Salt stress enhanced Cu/Zn-SOD activity in both cytosolic and chloroplastic fractions in Kharchia65, but there was little or no increase in the enzyme’s activity in mitochondria. Fe-SOD was predominantly active in the chloroplastic fraction. However, the enzyme was also found to be active in the cytosolic and mitochondrial fractions. Kharchia65 showed higher Fe-SOD and Cu/Zn-SOD activity than HD2687 under both normal and salt-stress conditions. In *Pisum sativum*, a salinity-induced increase in chloroplastic Fe-SOD was observed (Camejo et al., 2013).

Apart from salt stress, drought stress also causes a variety of physiological changes in plants, including a decrease in CO_2_ fixation due to abscisic acid-mediated stomatal closure, which leads to a reduction in photosynthetic rate and/or morphological abnormalities, mostly lower organ growth (Anjum et al., 2011). Furthermore, an increase in ROS production is associated with lipid peroxidation and alterations in enzymatic and non-enzymatic antioxidants (Csiszar et al., 2007; Tari et al., 2008; Dinakar et al., 2012). It has been found that total SOD activity appears to increase in the leaves of *Olea europaea* and the shoots of *Oryza sativa* cultivars when exposed to water stress (Sharma and Dubey, 2005; Sen and Alikamanoglu, 2013). However, an increase in total SOD activity was followed by a decrease in the number of leaves of *Gossypium* sp. and two grass species (*Festuca arundinacea* and *Poa pratensis*). On the flip side, a significant reduction of SOD activity was noticed for the pea plant, under drought stress (Deeba et al., 2012; Doupis et al., 2013). SOD activity enhanced or remained constant in *Triticum aestivum* during the early stages of drought but declined with chronic water stress (Deeba et al., 2012). Studies suggest that photosystem II can be protected by SOD from reactive O_2_ induced by oxidative and water stress (Deeba et al., 2012). In a recent study, the Tibetan wild *Hordeum vulgare* genotypes XZ16 and XZ5 were found to have considerably elevated SOD activity throughout the anthesis period when subjected to drought stress (Ahmed et al., 2013).

Cold stress is another major abiotic stress that can cause functional abnormalities in chloroplasts or mitochondria, resulting in ROS overproduction (Latef, 2021). Earlier research demonstrated that plants produce a lot of H_2_O_2_ during cold stress, and higher antioxidant enzyme activity tends to correlate with higher tolerance (Huang and Guo, 2005a). Studies in tolerant rice, barley, and tobacco cultivar roots and shoots revealed a differential amount of SOD activity under cold stress (Huang and Guo, 2005a; Xu et al., 2010; Mutlu et al., 2013). In sensitive as well as tolerant cultivars of tobacco the roots exhibited a higher SOD and catalase activity as compared to peroxidase activity (Xu et al., 2010). While tobacco shoots exhibited a higher peroxidase activity in comparison to SOD and catalase activity (Xu et al., 2010). In paraquat-treated *Nicotiana plumbaginifolia*, increased mRNA levels for mitochondrial Mn-SOD, chloroplastic Fe-SOD, and cytosolic Cu/Zn-SOD were observed in the presence of light. However, only cytosolic Cu/Zn-SOD expression was observed in the dark (Mylona et al., 2007). Further, convincing evidence was found for the stimulation of genes and enzyme activity in *Zea mays* embryos in response to paraquat. Benzyl viologen-produced O_2_ and O_2_^•^ was found to increase the expression of mitochondrial Mn-SOD and cytoplasmic Cu/Zn-SOD genes (Mylona et al., 2007). O_3_ (ozone) is a contaminant in the atmosphere that breaks down in the apoplast to produce primarily O_2_^•^ and H_2_O_2_. Acute or chronic exposure of *Zea mays* seedlings resulted in increased transcript levels of mitochondrial Mn-SOD and cytosolic Cu/Zn-SODs in the leaves (Rossa et al., 2002). On the other hand, the transcript level of chloroplastic Cu/Zn-SOD was shown to be down-regulated. The blue light wavelength has been shown to induce SOD activity more effectively in *Gracilariopsis tenuifrons* than other wavelengths (Rossa et al., 2002). UV B (280-350 nm) irradiation increased SOD activity in *Cassia auriculata* seedlings (Agarwal, 2007). Expression of Mn-SOD enzyme was also increased in *Nicotiana tabacum* leaves when exposed to high light intensity. However, increased total SOD activity was observed in *Hordeum vulgare* catalase-deficient mutants RPr 79/4 after the plants were exposed to high intensities of light (Azevedo et al., 1998; Dat et al., 2003).

#### Genetic engineering of plant SOD

The activation of SOD in response to various environmental conditions, as discussed in the previous section, suggests that it is a significant component of the plant’s defense system, and therefore, its genetic modification could lead to the development of stress-tolerant phenotypes. It was observed that *Oryza sativa* showed better resistance to methylmercury and drought stress when the *Pisum sativum* Mn-SOD gene was expressed in the chloroplasts (Wang et al., 2005). In another instance, overexpression of the Cu/Zn-SOD gene in *Nicotiana tabacum* plants was done to improve oxidative stress resistance, while tolerance to methyl viologen and pure cercosporin was achieved in sugar beet by transferring a *Lycopersicon esculentum* SOD gene (Tertivanidis et al., 2004; Wang et al., 2005). SOD genes have been shown to help plants eliminate ROS more efficiently under methyl viologen exposure or environmental stresses such as cold, ozone, water deficit, and salt stress (Wang et al., 2005). Nonetheless, there are several transgenic plants with SOD genes from different plants that did not show any improvement in stress tolerance (Tertivanidis et al., 2004; Sunkar et al., 2006). The differences in SOD isoenzymes, as well as the complexity of the ROS detoxification system, have been proposed as key variables for these instances (Kwon et al., 2002; Jia et al., 2009). When Mn-SOD was overexpressed in Arabidopsis, Mn-SOD, Cu/Zn-SOD, and Fe-SOD activity was higher in transgenic plants than wild-type plants, and the transgenic plants grew well and produced fewer lipid peroxidation products when treated with 150 mM NaCl (Mylona and Polidoros, 2010). The transcriptome of knockdown Arabidopsis plants with suppressed expression of chloroplastic Cu/Zn-SOD (CSD2) revealed the appearance of induced chloroplast and nuclear-encoded genes as a result of O_2_ buildup under optimal conditions (Mylona and Polidoros, 2010). Mn-SOD gene from halophilic archaeon was extracted and transferred into *Oryza sativa* by agrobacterium-mediated transformation to explore new gene resources for increasing *Oryza sativa* tolerance to salt stress (Wang et al., 2004). The transformants (L1 and L2) showed some MnSOD expression and increased overall SOD activity, resulting in improved ROS removal efficiency under salt stress. The amounts of oxygen and hydrogen peroxide (H_2_O_2_) were dramatically reduced; they also had better levels of photosynthesis and lower levels of relative ion leakage and malondialdehyde (MDA) concentration than wild-type plants (Wang et al., 2004). In contrast to the reports discussed above, overexpression of cytosolic or chloroplastic SOD in some transgenics only provided moderate or minimal tolerance, owing to the type of overexpressed SOD and its subcellular localization (Wang et al., 2004). Cu/Zn-SOD and FeSOD inactivation by H_2_O_2_, their reaction end product, and the competing resistance of Mn-SOD to H_2_O_2_ may be relevant to their specific effects. In this scenario, antisense technology is now feasible and should be used in conjunction with any overexpression research.

### Ascorbate peroxidase (APX) [EC 1.11.1.11]

#### Overview and types

**Figure 3:**
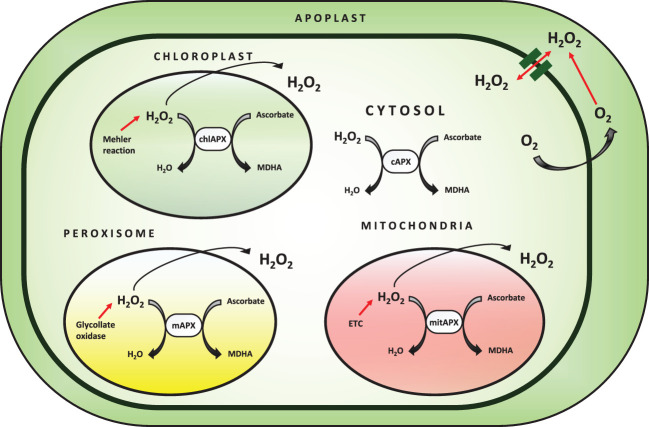
Subcellular localization of APX enzyme and detoxification of ROS.

Ascorbate peroxidase is a heme-containing peroxidase that catalyzes the oxidation of a wide spectrum of organic compounds in the presence of H_2_O_2_. APX is found in higher plants, chlorophytes, red algae, and members of the protist kingdom, and plays a crucial role in growth regulation (Wang et al., 2004; Chen et al., 2013). The peroxidase database contains APX and other peroxidase sequences from all kingdoms of life, as well as a set of bioinformatics tools for evaluating peroxidase sequences. Genomic and cDNA APX sequences have been discovered from a wide range of plants, demonstrating that APX is found throughout the vegetal domain. In these organisms, small gene families encode this enzyme (Oliva et al., 2009; Chen et al., 2013). The different isoforms of APX are categorized based on subcellular localization in the cell (**Figure 3**). Membrane-bound isoforms are present in microbodies (including peroxisome and glyoxysome) and chloroplast thylakoids, while soluble isoforms are found in the cytosol, mitochondria, and chloroplast stroma. The subcellular localization of the isoenzyme is determined by the presence of organelle-specific targeting peptides and transmembrane domains in the N and C-terminal regions of the protein (Teixeira et al., 2004; Passardi et al., 2007).

Furthermore, the plant chloroplastic APX isoenzyme-encoding genes are divided into two groups in plants. The first group consists of single genes that encode two isoenzymes. Genes from spinach (*S. oleracea*), tobacco (*N. tabacum*), pumpkin (*Cucurbita* sp), and ice plant (*Mesembryanthemum crystallinum*) are included in this group. Individual genes code for distinct isoenzymes that are separately regulated in the second category. Arabidopsis, rice, and tomato genes are included in this group. In spinach, the mechanism of alternative splicing in chlAPX was investigated, and the results revealed that alternative splicing is critical for modulating the expression of stromal (sAPX) and thylakoid (tAPX) isoenzymes, and this regulation happens in a tissue-dependent manner. In certain plant species, ascorbate peroxidases have been partially identified. The APX family in spinach is composed of genes that code for one cytosolic, two chloroplastic (sAPX and tAPX membrane) isoenzymes, one microbody membrane-targeted isoenzyme, and an unknown putative cytosol-soluble isoenzyme (Teixeira et al., 2006). Four cDNAs belonging to potential cytosolic, peroxisomal, and chloroplastic (thylakoid and stromal) APX isoforms were identified and studied in Cowpea (D’Arcy-Lameta et al., 2006). Six APX-coding loci were discovered in *Eucalyptus grandis*, with prediction tools indicating their subcellular localization. Three out of six isoforms were predicted to be cytosolic, one as a putative peroxisomal protein, and two as chloroplast-associated proteins (Teixeira et al., 2005). Another study in tomato revealed seven members where three were cytosolic, two peroxisomal, and two chloroplastic members (Najami et al., 2008). Two chloroplastic, one thylakoid-bound, and one membrane-bound, whose product is targeted to both chloroplast stroma and mitochondria, were identified in the model plant *Arabidopsis thaliana*, with the intracellular localization of an additional member still being unknown (Najami et al., 2008). Two chloroplastic, one thylakoid-bound, and one membrane-bound APX, whose product is targeted to both chloroplast stroma and mitochondria, were identified in the model plant *Arabidopsis thaliana*, while the intracellular localization of an additional member is still unknown (Najami et al., 2008). The APX gene family of rice has a total of eight members, with two members each in cytosol, peroxisome, chloroplast, and mitochondria. In rice, a novel protein called APX-R (Ascorbate peroxidase-related) has recently been discovered to be functionally connected with APX (Lazzarotto et al., 2011). Further analysis revealed that the Apx-R gene corresponds to a new class of heme peroxidases (Lazzarotto et al., 2011).

In the absence of AsA, APX isoenzymes are labile, and thus, a high quantity of endogenous AsA is required for the antioxidant system to effectively protect plants from oxidative damage (Maruta et al., 2010). The APX activity is quickly lost under particular conditions when the concentration of AsA is less than 20 μM, making chlAPX the least stable isoform. Both cAPX and mAPX have half-inactivation times of one hour or more, whereas mAPX and chlAPX have half-inactivation times of less than 30 seconds (Anjum et al., 2016).

#### Response of APX under different abiotic stresses

Drought stress, salt stress, high levels of light, high and low temperatures, pathogen attacks, H_2_O_2_, and abscisic acid all affect the expression of APX-producing genes (Teixeira et al., 2004; Passardi et al., 2007; Anjum et al., 2016). Furthermore, the transcriptional expression of APX genes varies depending on tissue and developmental stage (Teixeira et al., 2005). By restricting the amount of water available to the plant, salinity stress causes ion imbalances and physiological drought-like conditions. In such situations, APX provides varied levels of salt tolerance to affected plants. Salinity-induced oxidative damage is seen in cAPX mutants, but constitutive overexpression lines demonstrate increased resistance to 100 mM NaCl stress (Diaz-Vivancos et al., 2013). Tomato plants that overexpress pea cAPX are found to be more resistant to salinity stress (Wang et al., 2005). In transgenic Arabidopsis plants, overexpression of OsAPX2 exhibits higher tolerance to salt stress than OsAPX1. However, it is possible that the observed differences in tolerance are attributable to the positional influence of distinct transgenic lines. In transgenic tobacco, a cAPX from Arabidopsis displayed enhanced salt, drought, and PEG tolerance. Furthermore, in sensitive plants, salt stress causes lipid peroxidation and membrane damage, as well as low levels of antioxidant enzymes. Transgenic tobacco BY-2 cell lines with 50 and 75 percent decreased cAPX activity revealed higher ROS buildup. The study found that ascorbate peroxidase gene expression during stress resulted in salt and heat tolerance with no significant changes in levels of other ROS scavenging enzymes (Ishikawa et al., 2005; Wang et al., 2005; Nakano et al., 2006; Lu et al., 2007; Berwal et al., 2021).

Pea chloroplast APXs responded differently in saline conditions, with sAPX gradually increasing and tAPX gradually dropping, but a tAPX from *Solanum lycopersicum* introduced in tobacco gave enhanced resistance to salt and osmotic stress. Under salt stress, chlAPX activity increases, which protects against ROS generated in mitochondria and/or peroxisomes. Transgenic tobacco accumulating higher ascorbate was used to induce salt stress tolerance. Drought and salt conditions increased the expression of the APX gene in French bean seedlings (Eltelib et al., 2012; Nageshbabu and Jyothi, 2013). In Arabidopsis, overexpression of an APX from *Puccinellia tenuiflora*, a salinity-tolerant wild grass, boosted tolerance to 175 mM NaCl while also protecting against lipid peroxidation. Under salinity conditions, transcripts for mAPX from *Hordeum vulgare*, while a Populus peroxisomal APX in tobacco, conferred salt, drought, and MV stress resistance as well as larger roots (Li et al., 2009; Eltelib et al., 2012). Furthermore, APX mutants can upregulate other peroxidases to compensate for the loss of APX and give stress tolerance. This is validated by the expression of rice GPX in rice APX 1/2 mutants, as well as the upregulation of other enzymes (CAT and GP) in Arabidopsis APX3 knockout mutants that showed no indications of stress. When salicylic acid is administered topically, it stimulates APX and GR activity, resulting in increased salt tolerance in mung bean (Narendra et al., 2006; Bonifacio et al., 2011; Nazar et al., 2011; Guan et al., 2015; Husen et al., 2018). The response of all antioxidant enzymes to salt stress in Brazilian indica rice was studied at two developmental phases, and it was discovered that cAPX expression was up-regulated in 11-day-old seedlings but not in 6-week-old plants (Guan et al., 2015). This stress causes the production of ROS. The response of APX isozymes to this circumstance at different phases of plant growth is regulated. Under salinity stress, the normal salinity-resistant rice leaf basal region showed an increase in CAT and APX transcripts, whereas APX8 levels were slightly reduced. Under saline conditions, the expression of OsAPX2 did not change (Yamane et al., 2010). Another study found a similar drop in APX8 responsiveness when the other isoforms, APX2 and APX7, were strongly expressed during salt stress. In a different experiment, OsAPX8 demonstrated high expression in rice roots over a wide range of salt concentrations, from 150 to 300 mM, but OsAPX7 transcripts dropped dramatically at 300 mM. Age, cultivar, plant sections, and physiological conditions of plant growth all contribute to variances in APX gene expression (Teixeira et al., 2004; Hong et al., 2007; Passardi et al., 2007). Sweet potato plants with various levels of sensitivity to salt stress accumulated APX transcripts differently, with larger quantities in tolerant genotypes. APX plays a key function in plant drought resistance and recovery. Overexpressed P5CS gene in transgenic soybean and tobacco under drought stress elevate APX transcripts. In *Prunus* sp., APX and other Asc-GSH pathway enzymes were up-regulated during drought treatment and reduced throughout the recovery period (Sofo et al., 2005b; Teixeira et al., 2006; Zarei et al., 2012; Cruz et al., 2013). Studies have reported that glycine betaine (GB) increases APX under drought (Sofo et al., 2005b; Teixeira et al., 2006; Zarei et al., 2012; Cruz et al., 2013). Overexpression of cAPX (APX1) reduces drought symptoms, and transgenic tobacco plants outperformed non-transgenic plants. The relevance of this isoform in plant growth and development was also demonstrated by loss of function APX2 mutants, which were more vulnerable to drought than over-expression lines. Drought resistance was improved in over-expression lines of an mAPX from *Salicornia brachiata* relative to control plants (Zhang et al., 2013; Singh et al., 2014). cAPX is found to be important in drought stress tolerance in tobacco, with a primary benefit being membrane protection (Faize et al., 2011). Even under non-stressed conditions, APX activity is observed to be higher in tolerant cowpea plants. The sensitive cultivar up-regulates cAPX and mAPX in response to stress, whereas the tolerant cultivar up-regulates chlAPX (D’Arcy-Lameta et al., 2006). Various wheat genotypes have different levels of APX expression when there is a water crisis. cAPX1 was up-regulated in both genotypes, sAPX2 only in sensitive, and tAPX and cAPX2 only in tolerant cultivars (Secenji et al., 2009). After 15 days of drought stress in rice, the tAPX gene was down-regulated, while numerous other isoforms were upregulated. However, certain microsomal isoforms were only slightly or not at all altered (Rosa et al., 2010). This indicates that APXs are expressed differently in different species and under different conditions.

The temperatures that are either too low or too high have an adverse impact on plant physiology. Cold stress induces APX expression in tolerant maize lines, but not in sensitive ones (Caverzan et al., 2012). When compared to heat stress, low temperatures increase cAPX expression in potato tubers, implying that it plays a role in cold adaptation (Caverzan et al., 2012). Tobacco plants with a higher level of tAPX were more resistant to freezing and light stress, whereas Arabidopsis plants with a lower level of tAPX were more resistant to heat stress (Miller et al., 2007; Wang et al., 2016). Rice plants with homologous overexpression of a cAPX were tolerant to colder temperatures at the booting stage than wild-type plants, owing to enhanced APX activity in spikelets (Sato et al., 2011). SOD and APX gene expression in potato chloroplasts was induced using an inducible promoter SWPA2 that works under oxidative stress. With a substantial difference from the control, the plants obtained were tolerant to higher temperatures and methyl viologen (MV) stressors (Tang et al., 2006). In a similar experiment with sweet potatoes, tolerance to cold and MV stressors was observed (Lim et al., 2007). Transgenic plants with tomato tAPX expressed in tobacco were more resistant to both temperature and light stressors, and their photosynthetic efficiency was higher than non-transformed plants (Sun et al., 2010). High temperature increases cAPX in sweet potato leaves, but cAPX, mAPX, and sAPX were all up-regulated in cucumber after an initial decline (Park et al., 2004; Song et al., 2005). A cAPX has been reported to decrease quickly after heat shock treatment, negating its positive role in this stress, whereas some studies claim that APX2 is increased under heat circumstances (Gao et al., 2010). In Arabidopsis cells, APX1 is known to be active largely in response to heat and drought stress (Koussevitzky et al., 2008). In Arabidopsis, a mAPX from barley was overexpressed to display heat stress tolerance (Smeets et al., 2008). As a result, different APX isoforms and antioxidative mechanisms at multiple subcellular sites can be used to breed plants that can withstand environmental stress.

Heavy metal ion pollution in the soil is a major problem that reduces crop productivity. Under cadmium and arsenic stress, APX expression was induced in the leaves of *Arabidopsis thaliana, Solanum nigrum, and Brassica juncea*, but it was reduced in the leaves of *Brassica napus* (Smeets et al., 2008; Khan et al., 2009; Markovska et al., 2009; Nouairi et al., 2009; Pinto et al., 2009; Ansari et al., 2015). Copper stress increased APX expression in leaves of *Elsholtzia splendens*, however it was variable in *Withania somnifera* depending on metal ion concentrations (Peng et al., 2006; Khatun et al., 2008). The expression of APX isoforms in *Nicotiana tabacum* and *Typha angustifolia* leaves was observed to remain constant with varying levels of cadmium stress, while chromium and lead stresses did not cause any changes in APX expression in Typha leaves (Bah et al., 2011). Cadmium stress caused APX expression to vary in *Zea mays* (Ekmekçi et al., 2008). Low concentrations of cadmium stimulated APX activity in cells of coffee plant, but higher concentrations had no effect after 24 hours, while nickel enhanced APX activity at two extreme concentrations (Gomes-Junior et al., 2006). In rice, aluminium exposure causes practically all APX isoforms to become active. Double mutants of cAPX1/2 had a higher tolerance to high concentrations of aluminium (Sharma and Dubey, 2007; Rosa et al., 2010). This heavy metal enhances cAPX activity in pea at higher concentrations for longer periods of time, whereas it decreases and becomes constant beyond it (Panda and Matsumoto, 2010). De-rooted bean plants with inadequate cAPX were susceptible to iron, as were tobacco plants with deficient cAPX (Caverzan et al., 2012). Copper and cadmium increased APX activity in transgenic tall fescue plants compared to control, but arsenic decreased it in both transgenic and control plants (Lee et al., 2007). In *Eichhornia crassipes* seedlings, lead stress boosted APX activity (Lee et al., 2007). Cadmium chloride boosted APX activity in salt tolerant and sensitive rice cultivars, with the former having a higher activity (Roychoudhury and Ghosh, 2013; Malar et al., 2014). A similar increase in APX activity was seen in *Vigna radiata* (Roychoudhury and Ghosh, 2013). Salt and lead stress doubled on *Vigna radiata* seedlings resulted in an increase in APX activity (Siddiqui, 2013). As a result, several scientific publications have shown that APXs play a significant role in protecting plants from heavy metal stress in soil (Siddiqui, 2013).

### Catalases (CAT) [EC 1.11.1.6]

#### Overview

Catalases are tetrameric heme-containing enzymes that convert hydrogen peroxide to water and oxygen and are mostly found in peroxisomes (Srivalli et al., 2003). Catalase isozyme forms are found in many plants, including two in castor bean and six in Arabidopsis, and they can directly dismutate H_2_O_2_ or oxidize substrates such as methanol, ethanol, formaldehyde, and formic acid (Ben-Amor et al., 2005).

Plant catalases are divided into three groups based on their structures: class 1 catalases are found in photosynthetic tissues and are involved in the removal of H_2_O_2_ produced during photorespiration; class 2 catalases are found in vascular tissues and may play a role in lignification, though their exact biological role is unknown; and class 3 catalases are found in seeds and young plants, and their activity is linked to the removal of excess H_2_O_2_ produced during fatty acid degradation in the glyoxylate cycle in glyoxisomes (Ben-Amor et al., 2005). Catalases are the primary scavenging enzymes that may directly dismutate H_2_O_2_ and are required for ROS detoxification during stress (Ben-Amor et al., 2005). This is also related to the fact that during stress, peroxisomes proliferate, possibly aiding in the scavenging of H_2_O_2_ that diffuses from the cytosol (Ben-Amor et al., 2005). Increased catalase activity is thought to be an adaptive characteristic that could aid in overcoming tissue metabolic damage by lowering harmful levels of H_2_O_2_ (Mhamdi et al., 2010). In these organelles, a 200mM NaCl concentration resulted in a decrease in catalase activity (Srivalli et al., 2003). Increased catalytic activity in transgenic tobacco with sense cDNA of cotton catalase was shown to reduce photorespiratory loss, but antisense constructions reduced catalase specific activity, resulting in a commensurate increase in the CO_2_ compensation point. In alfalfa nodule, tea, cotton, and tobacco, abiotic stress causes upregulation of the genes responsible for catalase expression (Sekmen et al., 2007; Upadhyaya et al., 2008; Vital et al., 2008; Mhamdi et al., 2010). Catalases of class II have mostly been examined in relation to disease progression and resistance. It has been discovered that they are a target for SA (salicylic acid), and transgenic potato plants expressing the tobacco Cat2Nt gene could result in constitutive expression of the endogenous potato Cat2St gene and increased resistance to *Phytophthora infestans* (Vital et al., 2008). In dry and arid areas, two of the most common and frequent abiotic stresses are drought and salinity. Vegetation experiencing salt and drought stresses has evolved a range of physiological mechanisms to cope with harsh climatic circumstances (Zhang et al., 2008; Deeba et al., 2012). Abiotic stresses in semi-arid regions result in a loss of plant growth and productivity, which leads to several developmental, physiological, cellular, and molecular responses (Grover et al., 2011; Pinheiro and Chaves, 2011; Sobhanian et al., 2011). The majority of these responses are caused by photon intensity beyond the absorption capacity of stressed plants (**Figure 3**). Photorespiration is known to allow oxygenic photosynthesis by scavenging its most poisonous by-product, 2-phosphoglycolate, but it also causes substantial losses of freshly assimilated CO_2_ from most land plants (Rahman et al., 2023). Many studies have focused on the importance of the CAT catalysis pathway under drought and salt stress because of the critical involvement of CAT in photorespiration. Indeed, the persistence of CAT activity in drought-stressed plants’ leaves is likely responsible for the elimination of photorespiratory H_2_O_2_ generated when plants are subjected to higher levels of water deficit and salinity stresses. Photorespiration acts as an energy sink in these conditions, limiting photoinhibition and over-reduction of the photosynthetic electron transport chain (Bauwe et al., 2012). On this basis, photorespiration and the CAT pathway are no longer regarded as wasteful activities, but rather as critical and accessory components of photosynthesis and aspects of stress responses in green tissues for preventing ROS accumulation (De Pinto et al., 2013; Rahman et al., 2023). Drought stress and salt predispose the photosynthetic system to photoinhibition, leading to light-dependent inactivation of the principal photochemistry associated with photosystem II that often persists after rewatering (Deeba et al., 2012).

Indeed, decreased CO_2_ transport to the chloroplast and metabolic restrictions influence photosynthesis, which is one of the primary activities affected by water deficiencies and high salt concentrations (Grover et al., 2011; Pinheiro and Chaves, 2011). Due to the concurrent or even earlier suppression of growth, total plant carbon uptake is further reduced. Water deficiency, either directly or indirectly resulting in lower growth, has a significant impact on leaf carbohydrate status and hormonal balance. Increased levels of reactive oxygen species (ROS) such as superoxide anion (O_2_^-^), hydrogen peroxide (H_2_O_2_), and hydroxyl radicals are commonly related to plant adaptation to drought and salinity. ROS are by-products of aerobic metabolism, and their generation is boosted by the disturbance of the electron transport system and oxidizing metabolic processes in chloroplasts, mitochondria, and microbodies during stressful situations (Grover et al., 2011; Voss et al., 2013; Ramakrishna and Gill, 2018; Rajput et al., 2021). ROS are effectively eliminated by non-enzymatic and enzymatic antioxidants in non-stressful conditions, but during drought and saline conditions, ROS production surpasses the ability of antioxidative systems to remove them, resulting in oxidative stress (Vanderauwera et al., 2012; Nawaz et al., 2021). Catalase (CAT) isoforms are iron porphyrin enzymes that act as an efficient ROS scavenging system to protect cells from the oxidative damage caused by these two stressors (Mittler et al., 2011). Based on previous research, an increase in CAT activity is often connected to the degree of dryness that plants experience (Grover et al., 2011; Mittler et al., 2011). The root length increases gradually in *Panicum sumatrense* under drought stress at all growth stages, whereas the chlorophyll pigments and plant height decrease (Vanderauwera et al., 2012). According to the researchers, compatible solutes such as proline, glycine betaine, and free amino acid increased in all drought treatments (Ajithkumar and Panneerselvam, 2014; Nawaz and Wang, 2020). Furthermore, stress treatment increased the activity of antioxidant enzymes such as superoxide dismutase (SOD), catalase, and peroxidases, enabling this species to exhibit strong drought-tolerance characteristics. Leaf CO_2_ absorption rate and carboxylation efficiency characteristics decreased as the water deficit increased in another drought-tolerant species (*Jatropha curcas*). Leaf H_2_O_2_ level and lipid peroxidation were negatively and strongly linked with CAT activity in this species, indicating that drought-induced suppression of this enzyme could have a negative impact. Differences in antioxidant responses to drought in C3 and C4 plants are few, but they may be essential in understanding the metabolic antioxidant patterns of these two plant groups. Relative shoot growth rate, relative water content and osmotic potential, H_2_O_2_ content and nicotinamide adenine dinucleotide phosphate (NADPH) oxidase activity, CAT activity, CAT1 mRNA level, and lipid peroxidation were studied in *Cleome spinosa* (C3) and *Cleome gynandra* (C4) seedlings. Seedlings grown under control conditions consistently had higher antioxidant enzymes (excluding SOD) in *Cleoma spinosa* than in *Cleoma gynandra* (Ajithkumar and Panneerselvam, 2014). CAT activity was linked with CAT1 gene expression in *Cleoma spinosa*, but not with CAT1 gene expression in *Cleoma gynandra* for 10 days. Drought stress increased the levels and activity of the CAT enzyme in both species. The findings revealed that the antioxidant defense system in *Cleoma spinosa* was unable to limit the increased ROS generation under stress. The antioxidant system in *Cleoma gynandra*, on the other hand, was able to cope with ROS generation under drought stress, despite its induction being lower than in *Cleoma spinosa*. Ford et al. investigated the quantitative changes in protein abundance of three Australian bread wheat cultivars (*Triticum aestivum* L.) in response to drought stress using a series of multiplexed experiments (Uzilday et al., 2012). The three cultivars, namely Kukri (drought-intolerant), Excalibur (drought-tolerant), and RAC875 (drought-tolerant), were produced in the glasshouse with cyclic drought treatment that replicated field conditions. The proteome modifications in the three cultivars at different times during the water shortage period represented their physiological responses to drought. An increase in CAT and SOD isoforms, as well as a decrease in proteins involved in photosynthesis and the Calvin cycle, were seen in all three cultivars, indicating an increase in oxidative stress metabolism and ROS scavenging capacity, as well as ROS avoidance.

Using a transgenic wheat line, researchers evaluated the response of photosynthesis to drought, heat stress, and their combination in the same species (Ford et al., 2011). According to the study, all stresses reduced photosynthesis, but their combination amplified the negative impacts. For instance, drought stress was found to reduce the transpiration rate, stomatal conductance, and intercellular CO_2_ concentration. On the other hand, heat stress boosted these photosynthetic characteristics, but it also decreased antioxidant enzyme activity, and hence, the antioxidative defense system. Given the difficulty of examining biochemical and molecular responses in the field, where a variety of factors other than dryness play a crucial role, scientific work on CAT in tree species is uncommon. Olive plants were found to up-regulate the enzymatic antioxidant system under water deficient conditions (Wang et al., 2010). This reaction protects the cellular machinery and reduces ROS-induced cellular damage. In fact, CAT activity increased significantly in plant leaves subjected to drought stress. The significant increase in CAT activity found in leaves may protect chloroplasts, which are the principal generators and targets of ROS action and present persistent electron fluxes under stress conditions (Sofo et al., 2005a). Under drought conditions, the efficiency of autochthonous plant growth-promoting rhizobacteria (*Bacillus megaterium* (Bm), *Enterobacter* sp., *Bacillus thuringiensis*, and *Bacillus* spp.) was investigated in *Lavandula dentata* and *Salvia officinalis* (Foyer and Shigeoka, 2011). In these two plant species, each bacterium used various ways to ameliorate water constraint and drought stress, including CAT up-regulation. It was found that plant characteristics, such as a low shoot/root ratio and a high stomatal conductance, are essential determinants limiting bacterial effectiveness in increasing nutritional, physiological, and metabolic plant activities (Armada et al., 2014). Salinity in agricultural land is a serious concern worldwide, which puts severe constraints on crop growth and productivity in many places (Zhang et al., 2008; Tanou et al., 2012; Armada et al., 2014). High salinity causes water deficit and ion toxicity in many plant species, and their sensitivity to salt stress varies. Compatible solutes, such as proline, trehalose, and glycine betaine, are deposited at millimolar concentrations in transgenic plants under salt stress, acting as osmoprotectors in physiological responses and allowing the plants to better withstand soil salinity (Chen and Murata, 2011; Vanderauwera et al., 2011). Low levels of GB, administered exogenously, or created by transgenes for the production of suitable solutes, can also trigger the expression of stress-responsive genes, such as those responsible for scavenging reactive oxygen species (Vanderauwera et al., 2011). Furthermore, significant efforts have been made to investigate how genes react to salt stress in many organisms. The effects of NaCl on H_2_O_2_ content and CAT activity were investigated in various plants, including a single-celled alga (*Chlorella* sp.), an aquatic macrophyte (*Najas graminea*), and a mangrove plant (*Suaeda maritima*), which have a high tolerance to NaCl. When exposed to high levels of NaCl, all the examined plants produced considerable amounts of H_2_O_2_, and CAT activity rose dramatically in response to the NaCl treatment (Nounjan et al., 2012). Interestingly, the same investigators discovered that cultivating the plants in a high-salinity environment led to the generation of novel CAT isoforms. A gene encoding a small GTPase (MfARL1) from a subtracted cDNA library in *Medicago falcate* was identified to better understand the role of certain essential genes in the response to salt stress (Mallik et al., 2011).

Under salt stress, transgenic seedlings that constitutively express MfARL1 showed a higher survival rate. Salt stress significantly reduced chlorophyll concentration in wild-type plants, but not in transgenic plants. During these saline conditions, transgenic plants accumulated less H_2_O_2_ and showed less oxidative damage than their wild-type counterparts, which can be attributed to higher CAT activity. Peroxisomal CAT activity was found to be higher in tomato leaves and roots treated with various degrees of salt stress compared to controls, although CAT activity in pure leaf peroxisomes did not increase in response to salinity in other species such as peas (Wang et al., 2013). AtWNK8, mostly expressed in the primary root, hypocotyl, stamen, and pistil, appears to play a crucial role in salt and osmotic stress tolerance (Yang and Guo, 2018). Indeed, mutants overexpressing the WNK8 gene are more resistant to salt and osmotic stressors than the wild type (Yang and Guo, 2018). CAT activity in WNK8 mutants is higher than in wild-type plants under NaCl and sorbitol stress. This study provides evidence that the improved resistance of WNK8 mutants to salt stress is due to increased endogenous activities of CAT and GPX in association with increased proline synthesis and accumulation. Some plant pretreatments have been identified as effective ways to stimulate plant defenses against salt stress. For instance, the effects of two exogenous osmoprotectants (proline and trehalose) on a salt-sensitive rice variety reveal that salt stress caused growth reduction, an increase in the Na^+^/K^+^ ratio, an increase in proline level, and up-regulation of proline synthesis genes (pyrroline-5-carboxylate synthetase, P5CS) (Chen and Murata, 2011). Exogenous osmoprotectants did not appear to ameliorate growth inhibition during salt stress, but they appeared to have a significant favorable effect during the recovery period, with a larger percentage of growth recovery. The scientists discovered that an increase in CAT activity was linked to a considerable decrease in H_2_O_2_, especially in proline-treated plants. Administering proline to tree species (different wild almond species) can reduce the negative impacts of abiotic stresses like salinity, allowing leaves to better withstand oxidative stress by functioning as an effective H_2_O_2_ scavenger (Zhang et al., 2013). Furthermore, salt stress has been shown to cause considerable alterations in CAT activity in a variety of wild almond species (Sorkheh et al., 2012a). One study investigated the effects of H_2_O_2_ leaf spraying pretreatment on plant growth, and it was found that spraying H_2_O_2_ boosted antioxidant enzyme activity, with CAT being the most sensitive (Sorkheh et al., 2012b). Considering the protective effect of CAT, increased CAT activity appears to be linked to gene expression regulation, and decreased oxidative damage was identified in plants with higher CAT activity.

### Glutathione reductase (GR) [EC 1.8.1.7]

#### Overview

**Figure 4:**
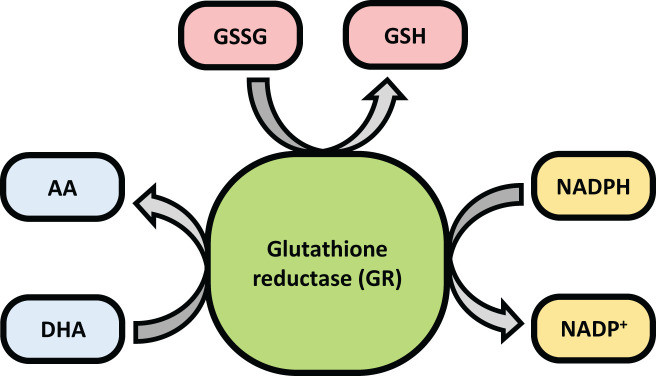
Diagram showing the role of glutathione reductase (GR).

**Figure 5:**
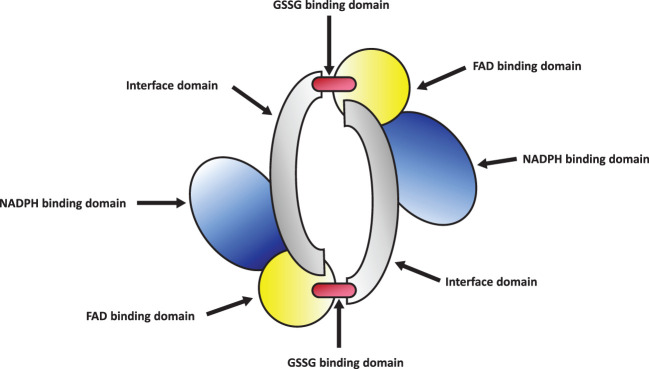
Structure of glutathione reductase homodimer.

Glutathione Reductase (GR or GSR) is a flavoprotein oxidoreductase that helps catalyze the reduction of glutathione disulfide (GSSG) to its reduced sulfhydryl form (GSH) using NADPH as a reductant. The reduced GSH formed is then utilized for the regeneration of ascorbic acid (AsA) using monodehydroascorbate (MDHA) and dehydroascorbic acid (DHA), thereby converting GSH to GSSG (**Figure 4**). GR has been shown to play a pivotal role in the plant defense against reactive oxygen metabolites generated by various abiotic stress conditions to which the plant is exposed (Gondim et al., 2012; Das and Roychoudhury, 2014; Aftab and Hakeem, 2022). Studies have described glutathione reductase as “a boon in disguise for abiotic stress defense operations”. GR efficiently maintains a relatively high cellular GSH/GSSG ratio by catalyzing the formation of a disulfide bond in glutathione disulfide (Gondim et al., 2012; Aftab and Hakeem, 2022). This enzyme is predominantly localized in the stroma of the chloroplast, but its isomers can also be found in mitochondria, cytosol, and peroxisomes (Gill et al., 2013). The enzyme is a homodimer of flavin adenine dinucleotide (FAD) having a molecular mass ranging from 100 to 150 kDa (**Figure 5**). An active site is located between the FAD binding domain and NADPH binding domain where the GSSG is bound (Ahmad et al., 2010). There is an additional interface region on each of the monomers of FAD that not only helps the GSSG to be bound between the subunits but also brings the FAD domains of each subunit in close proximity with the opposite catalytic site (Ahmad et al., 2010). It has been observed that in the absence of thiols, glutathione reductase has a tendency to form tetramers. However, GSH formed helps to maintain GR in its homodimeric configuration (Ahmad et al., 2010; Yousuf et al., 2012).

#### Catalytic activity of GR and response to abiotic stress

Glutathione reductase undergoes redox interconversion reactions (GSSG to GSH and GSH to GSSG) which depend on the availability of the required substrate (Rao and Reddy, 2008). For every mole of GSSG reduced to GSH, GR requires one mole of NADPH. The enzyme acts like a ping-pong mechanism where a hydride is transferred to FAD as the NADPH binds and it leaves before the di-glutathione binds (Rao and Reddy, 2008). The catalytic mechanism of GR has two phases. The first phase involves the reduction of the flavin moiety by NADPH. GR splits the 2 electrons provided by the reductant NADPH and donates the electrons to each of the two sulfur atoms of GSSG, one at a time. The second phase involves oxidation where the resulting dithiol reacts with GSSG and is reduced to 2 GSH at the active site of the enzyme (Rao and Reddy, 2008; Yousuf et al., 2012). The complete reaction can be represented as:


$$\text{GSSG}+\text{NADPH}\to 2\text{GSH}+\text{NAD}{\text{P}}^{+}(\text{by GR})$$

GR helps to regulate the GSH/GSSG ratio and supplies GSH to guaiacol peroxidase (GPX) and dehydroascorbate reductase (DHAR). GPX helps to remove H_2_O_2_ by combining GSH with H_2_O_2_ to form H_2_O and GSSG while DHAR reduces DHA using the GSH to form AA and GSSG (Dumanović et al., 2021).


$${\text{H}}_{2}{\text{O}}_{2}+\text{GSH}\to {\text{H}}_{2}\text{O}+\text{GSSG }(\text{by GPX})$$

DHA+GSH→AA+GSSG (by DHAR)

**Figure 6:**
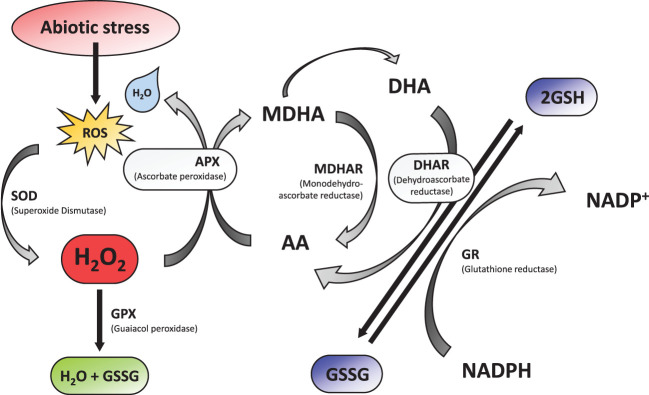
Diagram showing control of abiotic stress in plant GR.

GR catalyses the last rate limiting step of the Halliwell-Asada (AsA-GSH) pathway and is therefore linked with detoxification of ROS and abiotic stress tolerance in plants (Rao and Reddy, 2008; Hasanuzzaman et al., 2010; Hasanuzzaman et al., 2012). The maintenance of AsA and GSH reduced pools inside the cells is vital for ROS scavenging pathways and performing normal physiological activities. By converting GSSG to GSH, GR helps to maintain this equilibrium and thereby provide stress tolerance in plants (Rao and Reddy, 2008). Additionally, increased GR activity levels help increase the NADP^+^/NADPH ratio. Thus, the availability of NADP^+^ to accept electrons from the photosynthetic ETC is ensured, thereby minimizing the formation of ROS species (Mallik et al., 2011). A diagrammatic representation of abiotic stress control by GR is shown in **Figure 6**. Therefore, an implication of GR in transgenic plants can greatly reduce the ROS induced oxidative stress on the plant and enhance better plant development (**Table 2**).

**Table 2:** List of a few transgenic approaches implied for improved GR activity in plants.

Sl. No.	Transgenic plant	GR source	Response	Reference
1.	*Nicotiana tabacum* cv Samsun	*Escherichia coli* (gor)	Increase in GR activity by 2-10 folds	(Foyer et al., 1991)
2.	*Nicotiana tabacum* cv SR1	*Escherichia coli* (gor)	Increase in GR activity by 3 folds	(Aono et al., 1993)
3.	*Nicotiana tabacum* cv Samsun	*Pisum sativum* (gor1)	Increase in GR activity by 4.5 folds increase in overall GSH level	(Kornyeyev et al., 2001)
4.	Poplar hybrid (*Populus tremula x Populus alba*)	*Escherichia coli* (gor)	Chloroplasts were reported to show 1000 times higher GR activity; 2-10 times higher GR activity in cytosol; Transgenic plants with increased GR showed resistance to methyl viologen (MV)	(Sairam et al., 1998)
5.	*Brassica juncea*	*Escherichia coli* (gor)	A 2-fold increase of GR activity was noticed in cytosol while chloroplast showed 50 fold increase	(Hasanuzzaman et al., 2017)
6.	*Gossypium hirsutum* cv coker 312	*Arabidopsis thaliana* (GR)	Increased GR activity; Improved photochemical light utilization and decreased cold stress induced photo inhibition of PS II.	(Grover et al., 2011)

#### Response to drought stress and salinity

Shortages of water and high temperatures that lead to drought-like conditions have serious implications for the cellular machinery of a plant. Drought stress leads to impaired stomatal conductivity, slower rates of electron transport through the membrane transport chain, impaired CO_2_ diffusion levels, and reduced rates of photosynthesis. All these effects result in significant levels of ROS that cause extensive oxidative damage. Prolonged exposure to drought stress ultimately leads to reduced growth, resulting in lower crop yields (Grover et al., 2011). Several studies have shown an increase in GR activity when plants are exposed to drought stress (Hasanuzzaman et al., 2017). Water scarcity in *Ctenanthe setosa* results in a characteristic leaf-rolling adaptive response accompanied by increased GSH levels and decreased GSSG levels (Bian and Jiang, 2009; Saruhan et al., 2009). High GSH levels are known to be correlated with water content regulation in leaves (Jiang and Zhang, 2002). Studies have confirmed the effects of elevated GSH levels on drought stress tolerance and the reduction of damages induced by ROS (Bian and Jiang, 2009). GR helps to reduce GSSG to GSH in the presence of NADPH and maintains the reduced GSH pool inside the cell, thereby playing a significant role in stress tolerance. Numerous studies have confirmed the elevated levels of GR activity during drought stress in plants, including barley, maize, wheat, and rice (Kocsy et al., 2002; Chen et al., 2004; Chinnusamy et al., 2005; Selote and Chopra, 2006). A primary effect of drought stress is osmotic stress leading to a sudden change in the solute concentration around the cell and a rapid efflux of water from inside the cell. Kocsy et al. observed that osmotic stress resulted in an increase in GSH levels in wheat seedlings and GR activity in maize (Sumithra et al., 2006). With water scarcity, salinity levels also increase. Saline conditions result in osmotic inhibition and ionic toxicity, affecting normal physiological functions (Demiral and Turkan, 2005; Huang and Guo, 2005b). An increase in GR activity during salinity stress was reported in pea, cantaloupe, soybean, rice, tomato, *Arabidopsis thaliana*, and wheat (Szalai et al., 2009; Repetto et al., 2012; Hasanuzzaman et al., 2013). These results provide conclusive evidence that GR plays a key role during drought and salt stress in plants.

#### Response to extreme temperatures

Extremes of temperature, both high and low, are major factors that contribute to poor crop yield and overall plant development. Higher temperatures result in overproduction of ROS, which leads to increased lipid peroxidation, inactivation of the oxygen evolving complex, membrane damage, and DNA damage (Nahar et al., 2015). Similarly, extreme low temperatures also lead to overproduction of ROS due to membrane fluidity degradation, impaired photosynthetic activity, and improper ROS detoxification (Zhang et al., 2008). This highlights the importance of the GSH pools and GSH redox state as vital components in plant thermotolerance. In some maize varieties, GR activity was reported to increase greatly under high temperature (HT) stress treatment (Sumithra et al., 2006). Elevated levels of GSH in wheat at high temperatures suggest the role of GR in thermotolerance (Nahar et al., 2015a). Similar findings were reported in maize and *Vigna radiata* (Payton et al., 2011). Elevated GSH levels in mustard seedlings suggest efficient eradication of H_2_O_2_, thereby confirming increased GR activity (Kuk et al., 2003). High levels of GSH and GR activity were also reported in apple during the reproductive stages, further suggesting their enhanced roles in thermotolerance (Kuk et al., 2003). Low ambient temperature limits the activity of enzymes in the Calvin Cycle, disrupting the sulfhydryl groups and reducing CO_2_ assimilation (Zitka et al., 2013). Restricted carbon metabolism in the Calvin Cycle leads to insufficient supplies of electron acceptors and overproduction of ROS (Voss et al., 2013). Several studies have shown a positive correlation between cold stress and increased GR activity, including French bean seedlings, rice, and eastern white pine (Peuke and Rennenberg, 2005a; Hasanuzzaman and Fujita, 2013).

#### Response to heavy metal toxicity

Heavy metals are required for various plant processes and development, but excess heavy metal concentration can lead to toxicity (Peuke and Rennenberg, 2005a). Rapid industrialization has increased heavy metal concentrations in the environment beyond natural sources, disrupting normal physiological growth and generating ROS and oxidative damage (Peuke and Rennenberg, 2005b). To reduce damage and restore normalcy, plants activate various anti-oxidative defense responses, including phytoremediation (Jablonkai, 2022). GSH protects the plant cellular machinery against ROS-oxidative damage in three potential ways: 1) direct quenching of ROS; 2) conjugation of heavy metals and xenobiotic agents to GST; and 3) acting as a precursor for the synthesis of phytochelatins (PCs). By maintaining high levels of PCs, plants can withstand heavy metal stress. GR plays a key role in plant tolerance against heavy metals. GSH is a pivotal factor in the rate-limiting step for phytochelatin formation. The phytochelatins produced form complexes with various heavy metal ions and are sequestered to the vacuole for degradation, thereby limiting oxidative damage (Nahar et al., 2016). Reduced GSH levels are constantly monitored by GR and play a key role in heavy metal stress tolerance. Elevated levels of GR have been reported in Cd-induced stress, and its role in detoxification of ROS via the AsA-GSH cycle has been reported in plants such as radish, soybean, sugarcane, and *Arabidopsis thaliana*.

## Conclusion

Abiotic stresses pose a great challenge for plant growth and development by causing physiological, morphological, and biochemical changes in plant cells. The most common manifestation of abiotic stress is the production of ROS. ROS is both a harmful and beneficial molecule. At low or moderate concentrations, it mediates signal transduction that assists in maintaining cellular homeostasis and facilitates plant acclimatization to stresses. However, its overproduction causes significant damage to plant cells, such as lipid peroxidation, DNA damage, etc. The mechanism for maintaining equilibrium between ROS generation and their quenching involves the production of both enzymatic and non-enzymatic antioxidants. In the last two decades, significant progress has been made in effective ROS scavenging through genetic engineering approaches towards the development of stress-resilient crops. Furthermore, there is a pressing need to identify the genes and understand their mechanisms in the regulation of ROS signaling pathways. Knowledge about the genes and their mechanism of action will definitely help enhance abiotic stress resistance under real agricultural field conditions.

## Author contributions

NM and GS provided the fundings. NM and CJ wrote the article. LC and AP gathered the data. AC polished the article.
